# Structural covariance alterations reveal motor damage in periventricular leukomalacia

**DOI:** 10.1093/braincomms/fcae405

**Published:** 2024-11-12

**Authors:** Jieqiong Lin, Xin Zhao, Xinxin Qi, Wen Zhao, Songyu Teng, Tong Mo, Xin Xiao, Peng Li, Turong Chen, Guojun Yun, Hongwu Zeng

**Affiliations:** Department of Radiology, Shenzhen Children’s Hospital, Shenzhen 518038, Guangdong Province, China; China Medical University, Shenyang 110122, Liaoning Province, China; China Medical University, Shenyang 110122, Liaoning Province, China; Shantou University Medical College, Shantou 515041, Guangdong Province, China; China Medical University, Shenyang 110122, Liaoning Province, China; Department of Radiology, Shenzhen Children’s Hospital, Shenzhen 518038, Guangdong Province, China; Department of Radiology, Shenzhen Children’s Hospital, Shenzhen 518038, Guangdong Province, China; Department of Radiology, Shenzhen Children’s Hospital, Shenzhen 518038, Guangdong Province, China; Department of Rehabilitation, Shenzhen Children’s Hospital, Shenzhen 518038, Guangdong Province, China; Department of Rehabilitation, Shenzhen Children’s Hospital, Shenzhen 518038, Guangdong Province, China; Department of Radiology, Shenzhen Children’s Hospital, Shenzhen 518038, Guangdong Province, China; Shantou University Medical College, Shantou 515041, Guangdong Province, China

**Keywords:** periventricular leukomalacia, voxel-based morphometry, cerebral palsy, structural covariance, graph theory

## Abstract

Periventricular leukomalacia is a common neuroimaging finding in patients with spastic cerebral palsy. Myelin damage disrupts neuronal connectivity. However, specific alterations in the grey matter structure and their impact on the whole brain remain unclear, particularly when differentiating between preterm and full-term periventricular leukomalacia. This study investigated the grey matter network alterations following early white matter injury in infants and young children. High-resolution T_1_-weighted 3 T brain magnetic resonance imaging, clinical data and motor function scores were collected from 42 children with periventricular leukomalacia and 38 age- and sex-matched healthy controls. Based on gestational age, the periventricular leukomalacia group was stratified into preterm (*n* = 27) and full-term (*n* = 15) groups. Voxel-based morphometry was used to analyse whole-brain structural metrics, and motor-related regions were selected as nodes for network construction. Structural covariance analysis was used to quantify the strength of the structural connections between grey matter regions, and graph theory metrics were used to assess network properties. Motor assessments included gross and fine motor skills, and their associations with brain regions were analysed. Both preterm and full-term periventricular leukomalacia groups exhibited abnormal motor networks. Preterm periventricular leukomalacia showed more extensive central grey matter nuclei atrophy, whereas full-term periventricular leukomalacia was predominantly localized to the motor cortex. Children with periventricular leukomalacia displayed decreased connectivity between the central grey matter nuclei and other regions, coupled with increased connectivity between the motor cortex and cerebellar hemispheres. Thalamic volume correlated with gross motor scores in preterm infants. These findings suggest that ischaemic–hypoxic injury disrupts motor grey matter networks, with preterm infants being more severely affected. This study highlights the potential of structural covariance patterns for monitoring brain development and advancing our understanding of aberrant brain development in children with periventricular leukomalacia.

## Teaser

Lin *et al*. conducted a quantitative analysis of MRI structural data to investigate the alterations and differences in motor neural networks between preterm and full-term children with periventricular leukomalacia. The findings revealed more extensive neural network damage in preterm children and confirmed a correlation between thalamic volume and motor scores.

## Graphical Abstract

**Graphical Abstract:**
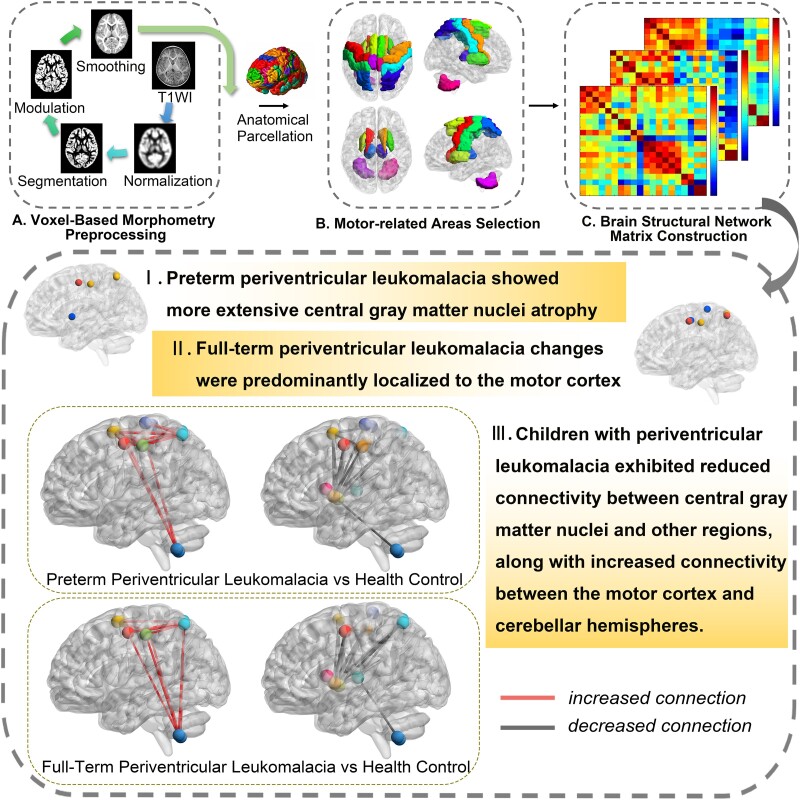

## Introduction

Periventricular leukomalacia (PVL) is a leading cause of cerebral palsy (CP), accounting for 40–50%^1,2^ of CP cases in infants with CP. PVL is characterized by white matter (WM) necrosis, microglia activation, NF-kβ upregulation and neuronal death.^3^ It primarily affects preterm infants because of their increased susceptibility to ischaemic injury caused by the fragile regulation of the small arteries supplying the periventricular WM, leading to transient haemodynamic disturbances. Although PVL can also occur in full-term infants, most current neuroimaging studies have focused on preterm PVL (pPVL), leaving the mechanisms underlying brain injury in full-term PVL (fPVL) relatively unexplored.

The clinical presentation of pPVL and fPVL differs significantly. pPVL often presents with diplegia, whereas fPVL commonly results in hemiplegia. Additionally, children with fPVL generally show better motor outcomes, with 80% achieving independent walking compared with only 46.1% of preterm infants. Conventional MRI studies have shown that full-term infants tend to have more asymmetric lesions and less extensive intracerebral involvement,^3^ suggesting a distinct pathophysiological mechanism between pPVL and fPVL. However, the specific effects of early ischaemic–hypoxic injury are poorly understood.

Animal studies have indicated that the WM of preterm newborns is more susceptible to oxidative damage than that of term infants.^4^ Moreover, WM injury in newborns has been linked to impaired growth and maturation of grey matter (GM). Research has identified damage to GM nuclei, such as the thalamus, in children with pPVL, which correlates with cognitive deficits.^5-8^ Autopsy studies have further revealed neuronal and axonal damage across various brain regions in 30–40% of PVL cases, with the thalamus, pallidum and dentate nucleus of the cerebellum exhibiting the most severe neuronal loss.^9,10^

Whether disturbances in GM growth result directly from WM injury or from independent mechanisms of neuronal immaturity remains unclear. However, early disruption of myelin development may affect neuronal connectivity during crucial phases of brain development.^4,11^ The GM plays an important role in the motor development and regulation of infants. A large cohort study linked early developmental disruptions caused by preterm birth to altered functional connectivity and noted abnormal increases in connections in the parietal superior lobule of the lateral motor network, which may contribute to developmental coordination disorders in preterm infants.^12^

Structural covariance analysis is a valuable tool for exploring the complex network mechanisms of the human brain by examining the covariance of morphological markers. This method assesses the relationship between the morphology of different brain regions and their structural and functional connectivities. Recent advancements in structural covariance methods have facilitated the mapping of myelin and glucose metabolism networks^13,14^ and integration of multiple anatomical indices from various neuroimaging modalities.^15,16^

Considering these findings, we hypothesized that the severity of ischaemic–hypoxic injury differs between preterm and full-term infants, leading to distinct long-term motor outcomes. Given the critical role of neuronal connectivity in early brain development, our study focused on alterations in the structural network of the GM. We employed voxel-based morphometry to correlate brain regions with motor scores and to identify key areas associated with movement modulation, analysed the volumes of motor-related brain regions using covariance analysis and compared the motor networks of preterm and full-term children with PVL using graph theory approaches. Our aim was to elucidate the impact of ischaemic–hypoxic injury on brain structural networks in children.

## Materials and methods

### Study participants

We prospectively enrolled patients aged 1–8 years with PVL-associated CP from the Rehabilitation Department of Shenzhen Children’s Hospital between 2019 and 2022.

For CP with PVL, the inclusion criteria were as follows: (i) confirmed CP diagnosis through medical history and neurological examination and (ii) evidence of PVL sequelae on conventional MRI performed during childhood. The exclusion criteria were as follows: (i) non-progressive dyskinesia since age ≥3 years; (ii) dyskinesia is caused by various factors (psychiatric disorders, tumours, metabolic abnormalities, genetic disorders, muscle atrophy, spinal cord lesions, muscle/peripheral nerve disease and hypotonia); (iii) contraindications to MRI (metal implants, severe hyperthermia, claustrophobia, critical conditions requiring life support systems); and (iv) inability to cooperate or excessive motion artefacts during MRI.

Age- and sex-matched children without growth retardation who underwent routine examinations in other clinical departments served as healthy controls (HCs). The study was approved by the Ethics Committee of Shenzhen Children’s Hospital (project no. 202004105), and all participants provided written informed consent.

### Clinical evaluation

Baseline demographic and clinical data, including age, gestational age at birth, birth weight, lesion laterality, PVL grade, CP type and perinatal history, were collected. Motor dysfunction severity was assessed using the gross motor function measure (GMFM) and the fine motor function measure (FMFM).

### MRI acquisition

MRI data were acquired on the 3.0 T Siemens Skyra system with a paediatric head coil, using 3D-MPRAGE sequences (parameters: echo time/repetition time = 2.26/2300 ms; field of view = 256 mm × 200 mm; slice thickness = 1.0 mm; spacing = 0.5 mm; flip angle = 8°; voxel size = 1.0 × 1.0 × 1.0 mm^3^; 175 slices).

### Voxel-based morphometry analysis

**Figure 1:**
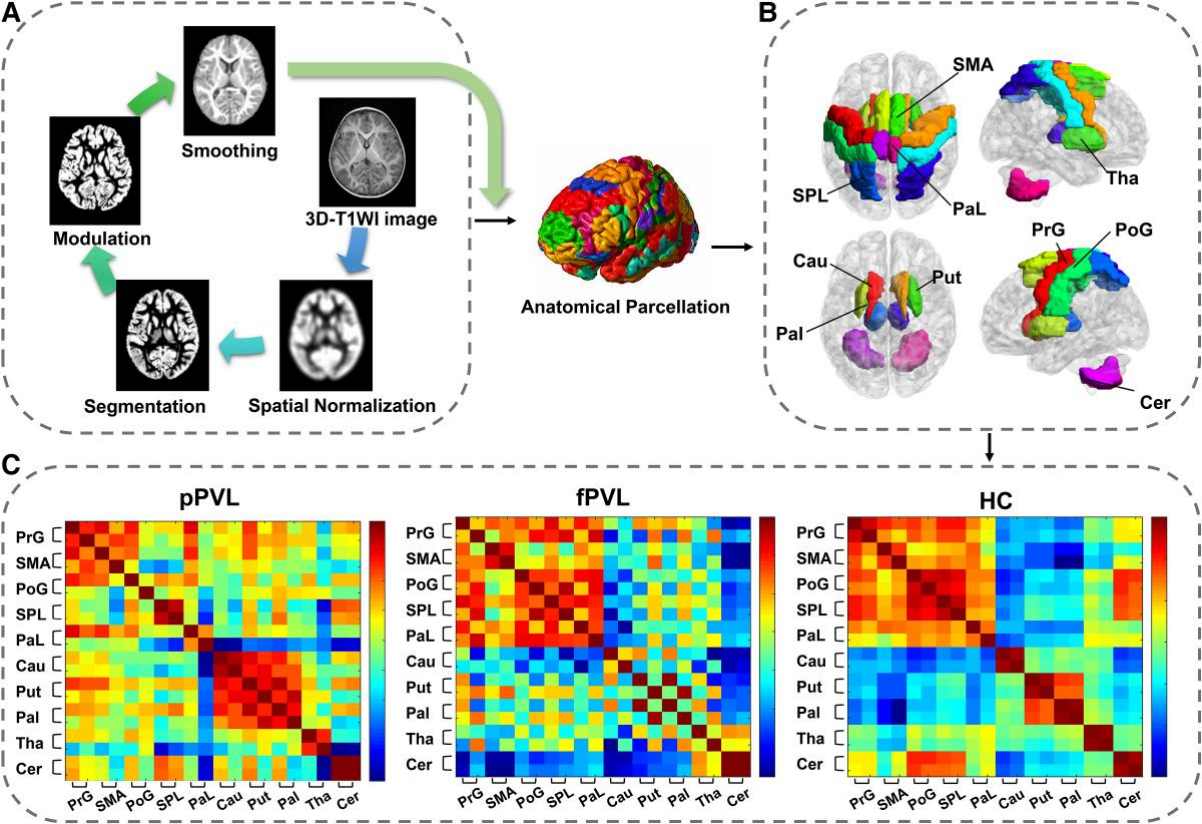
**Voxel-based morphometry (VBM) and structural network analysis**. (**A**) VBM morphometry preprocessing for the participants (*n* = 80). (**B**) Ten pairs of brain regions related to motor controlling were selected as nodes of interest: bilateral precentral gyrus (PrG), supplementary motor area (SMA), postcentral gyrus (PoG), superior parietal lobule (SPL), paracentral lobule (PaL), caudate (Cau), putamen (Put), pallidum (Pal), thalamus (Tha) and cerebellum (Cer). (**C**) Group mean Fisher’s z-transformed correlation coefficient matrices representing all ROI pairs in the pPVL (*n* = 27), fPVL (*n* = 15) and HC (*n* = 38) groups. The red–blue colour gradient represents the strength of the correlation between two nodes.

MRI data were preprocessed and analysed using MATLAB R2019a (The MathWorks Inc., Natick, MA, USA) and Statistical Parametric Mapping software (SPM12; The Wellcome Centre for Human Neuroimaging, UCL Queen Square Institute of Neurology, London, UK; www.fil.ion.ucl.ac.uk/spm). T_1_-weighted images were checked by a radiologist to eliminate those with possible artefacts or gross anatomical abnormalities that could affect preprocessing. All images were aligned along the anterior–posterior commissure and processed using Diffeomorphic Anatomical Registration Through Exponentiated Lie Algebra, implemented in the SPM12 toolbox. Segmentation was performed on each structural image, and the resulting GM and WM images were used to generate an unbiased study-specific template. All data were normalized to the Montreal Neurological Institute 152 (MNI-152) space using this template to account for residual normalization inaccuracies and anatomical variations. The GM images were smoothed with an 8-mm isotropic Gaussian kernel following visual inspection for homogeneity across the sample (Fig. 1A). Modulated, smoothed and normalized images were used for statistical analysis. In addition, the total intracranial volume (TIV), global GM and global WM were measured for all participants using maps derived from the unified segmentation of the high-resolution T_1_-weighted image.

Total GM and WM volumes (ml) were obtained using SPM 12 segmentation. TIV, GM and WM were calculated. Group comparisons for these global structural variables were conducted using analysis of covariance, controlling for age, and *post hoc t*-tests were used to examine differences between specific subtypes using SPSS.

Two-sample *t*-tests were performed to compare the regional GM volumes between the PVL and HC groups. For further subgroup analysis, an SPM full factorial analysis was employed, followed by *post hoc t*-tests, with the independent variables being the subgroups (pPVL, fPVL and HC).

The analysis was based on whole-brain structural data, with the age, sex and TIV of each participant as covariates. An absolute threshold masking of 0.1 was applied, and statistical significance was set at the voxel level [*P* < 0.0001, family-wise error (FWE)-corrected], with a cluster threshold of 100 voxels.

### Statistical analysis

The sample size was calculated using G*Power software (https://www.psychologie.hhu.de/arbeitsgruppen/allgemeine-psychologie-und-arbeitspsychologie/gpower). We set a desired power of 0.80 and an alpha error of 0.05, assuming an effect size of 0.40. The minimum sample size required to detect a significant difference between groups was 66. To compare demographic data between groups, a *t*-test or analysis of covariance was performed for continuous variables and χ^2^ tests for categorical variables using SPSS (version 20.0; IBM Corp., Armonk, NY, USA).

A general linear model was used to explore the potential relationship between the brain volume and motor scores. Regions of interest (ROIs; left and right thalamus) were chosen based on the automated anatomical labelling atlas created using the Wake-Forest University PickAtlas tool 3.0. Regression coefficients were determined by drawing 3.5-mm spherical ROIs around the peak voxel of significant clusters using MarsBar (http://marsbar.sourceforge.net). Mean GM estimates were extracted to conduct a correlation analysis with the respective motor scores using SPSS. TIV, age and sex were included as nuisance regressors. Results were considered significant for an α of *P* < 0.05 (FWE-corrected for multiple comparisons).

A series of nodes (*n* = 20), including the bilateral precentral gyrus, supplementary motor area, postcentral gyrus, superior parietal lobule, paracentral lobule, caudate, putamen, pallidum, thalamus and cerebellum, representing the brain ROIs for connectivity analysis were selected. Volumes were extracted from each ROI to create subject × ROI matrices (Fig. 1B). Graph analysis toolbox^17-19^ (http://www.nitrc.org/projects/gat/) was used to examine the motor covariance network of the participants.

For each group (pPVL, fPVL and HC), a motor covariance network was constructed with nodes representing brain regions (individual motor structure volumes) connected by edges representing connections (calculated as correlation coefficients between each pair of brain regions, controlling for age, sex and TIV; Fig. 1C).

To improve normality, the correlation coefficients underwent an r-to-z transformation. Each group was presented with an upweighted, undirected connection matrix that quantified the connectivity strength (structural covariance) between every pair of regions.

Using two-sample *t*-tests, the null hypothesis of equality in structural covariance between the patient and control groups was evaluated for each of the 20 pairs of regions. Network-based statistics were applied to control the FWE rate across the 20 multiple comparisons, with a *t*-statistic threshold of 3.1 and an FWE rate threshold of 5%. Network-based statistic^20^ (http://www.nitrc.org/projects/nbs/) was performed independently for each group.

Permutation testing was performed to determine whether the structural covariance was significantly stronger (or weaker) between regions related to motor control compared with randomly selected groups of regions. The mean structural covariance was calculated using 5000 sets of randomly chosen nodes. The connectivity strength within the motor network (cortex–basal ganglia nuclei–cerebellum) was compared with that between randomly chosen regions.

Graph theory was utilized to calculate various network measures, including global and local efficiency, assortativity, clustering coefficient, shortest path length, small worldness index, nodal local efficiency, nodal degree centrality, nodal betweenness centrality and nodal clustering coefficient. Detailed computation of network properties based on graph theory is provided in Supplementary material. Hubs are nodes occupying a central position in the overall organization of a network, identified as nodes with a participation coefficient that is 1 SD higher than the mean degree centrality.

Network measures were calculated across varying network density levels (0.1–0.5 with a step size of 0.01) and then averaged. Non-parametric permutation tests with 1000 permutations were performed to test statistical significance (*P* < 0.05), employing the false discovery rate for multiple corrections when analysing local connectivity changes.

## Results

### Demographic and clinical characteristics

Of the 85 participants who underwent MRI, 80 were included in the final analysis after excluding five for poor image quality. The clinical characteristics of the participants are summarized in Table 1. The median age of participants was 2.96 years. The groups did not differ in age, sex or *in vitro* fertilization rate. However, the pPVL group had lower birth weight (pPVL versus HC: 1.82 ± 0.87 versus 3.35 ± 0.36; *P* < 0.001) and higher caesarean delivery rate (pPVL versus HC: 66.7% versus 26.3%; *c*^2^ = 10.481; *P* = 0.001) than the HC group. In this study, all patients with PVL were diagnosed with CP. Among them, 15 patients exhibited spastic hemiplegia, 18 had spastic diplegia, 5 presented with spastic quadriplegia and 4 had dyskinetic CP. Moreover, the fPVL group had a higher incidence of hemiplegia (pPVL versus fPVL: 14.8% versus 73.3%; *c*^2^ = 14.383; *P* < 0.001), whereas the pPVL group had a higher incidence of diplegia (63.0% versus 5.6%; *c*^2^ = 12.479; *P* < 0.001) and bilateral PVL (88.9% versus 26.7%; *c*^2^ = 16.800; *P* < 0.001; Table 1).

**Table 1:** Demographic and clinical characteristics

	PVL (*n* = 42)	HC (*n* = 38)	PVL versus HC (*P*-value)	pPVL (*n* = 27)	fPVL (*n* = 15)	pPVL versus fPVL versus HC (*P*-value)
Age/years, [M (Q25, Q75)]	2.67 (1.83, 3.75)	3.34 (2.75, 4.42)	0.104	2.75 (2.00, 3.75)	2.58 (1.50, 3.34)	0.079
Sex, male/female	25/17	22/16	0.882	15/12	10/5	0.774
BW/kg, mean (SD )	2.35 (1.04)	3.35 (0.36)	<0.001***	1.82 (0.87)	3.31 (0.50)	<0.001***
Gestational age at birth, mean (SD )	35.74 (4.47)	37.33 (3.31)	0.26	33.65 (3.70)	40.25 (1.91)	<0.001***
Caesarean delivery, *n* (%)	25 (59.5%)	10 (26.3%)	0.003**	18 (66.7%)	7 (46.7%)	0.005**
IVF, *n* (%)	3 (7.1%)			2 (7.4%)	1 (6.7%)	
Paralysis type						
Hemiplegia, *n* (%)	15 (35.7%)			4 (14.8%)	11 (73.3%)	<0.001***
Diplegia, *n* (%)	18 (42.9%)			17 (63.0%)	1 (5.6%)	<0.001***
Quadriplegia, *n* (%)	5 (11.9%)			3 (11.1%)	2 (13.3%)	1.000
Dyskinetic, *n* (%)	4 (9.5%)			3 (11.1%)	1 (6.7%)	1.000
Symptoms						
Motor dysfunction, *n* (%)	42 (100%)			27 (100%)	15 (100%)	
Cognitive dysfunction, *n* (%)	15 (35.7%)			10 (37.0%)	5 (33.3%)	0.810
Language dysfunction, *n* (%)	13 (31.0%)			9 (33.3%)	4 (26.7%)	0.921
Epilepsy, *n* (%)	4 (9.5%)			3 (11.1%)	1 (6.7%)	1.000
Neuroimage findings						
Unilateral PVL, *n* (%)	15 (35.7%)			3 (11.1%)	11 (73.3%)	
Bilateral PVL, *n* (%)	27 (64.3%)			24 (88.9%)	4 (26.7%)	
PVL grade						
Mild, *n* (%)	7 (16.7%)			1 (3.7%)	6 (40.0%)	
Moderate, *n* (%)	23 (54.8%)			18 (66.7%)	5 (33.3%)	
Severe, *n* (%)	12 (28.6%)			8 (29.6%)	4 (26.7%)	

BW, birth weight; IVF, *in vitro* fertilization; M, median; Q25, the 25th percentile; Q75, the 75th percentile; ***P < 0.001; **P < 0.01.

### GMFM and FMFM assessment

Significant group differences were found in the total GMFM score, difficulty value, GMFM B area score, GMFM C area score, GMFM D area score and GMFM E area score between the PVL and HC groups.

The pPVL group exhibited lower GMFM total score, difficulty value, GMFM B area score, GMFM C area score, GMFM D area score, GMFM E area score, FMFM total score, ability value, FMFM D area score and FMFM E area score than the HC group. Similarly, the fPVL group showed lower GMFM total score, difficulty value, GMFM B area score, GMFM C area score, GMFM E area score, FMFM B area score and FMFM C area score than the HC group. No significant differences were found in the GMFM or FMFM scores between the pPVL and fPVL groups (Supplementary Table 1, Supplementary Fig. 1).

### Voxel-based morphometry analysis

The pPVL group had significantly smaller TIV (HC versus pPVL; 1347.77 ± 25.32 versus 1156.37 ± 28.81; *P* = 0.015), GM volume (HC versus pPVL; 740.65 ± 12.92 versus 633.74 ± 14.71; *P* = 0.004) and WM volume (HC versus pPVL; 399.24 ± 8.34 versus 302.15 ± 9.49; *P* < 0.001) than the HC group, after controlling for age. No significant differences were found between the fPVL and HC groups or between the pPVL and fPVL groups in terms of TIV, GM volume and WM volume.

After controlling for covariates, the PVL group showed significantly smaller bilateral thalamic volumes than the HC group (*P* < 0.0001, FWE-corrected). *Post hoc* analysis revealed that this difference was primarily driven by the pPVL group, which showed significantly smaller bilateral thalamic volumes (*P* < 0.0001; FWE-corrected) than the HC and fPVL groups. The volume of the left thalamus in the fPVL group was smaller than that in the HC group (*P* < 0.0001; FWE-corrected). No differences were found between the pPVL and fPVL groups in regional GM volumes (Table 2).

**Table 2:** Regions of significant volume differences between the PVL and HC groups (HC > PVL)

Anatomical region	MNI coordinates			Voxel	*Z* value	T/F	*P*-value uncorrected	*P*-value FWE-corrected
	*X*	*Y*	*Z*					
PVL versus HC								
Left thalamus	−29	−21	29	2460	5.97	6.78	<0.0001	<0.0001
Right thalamus	27	−33	30	2298	5.44	6.04	<0.0001	<0.0001
pPVL versus HC								
Left thalamus	−14	−21	9	2446	7.55	138.6	<0.0001	<0.0001
Right thalamus	17	−23	15	2342	5.95	48.01	<0.0001	<0.0001
fPVL versus HC								
Left thalamus	−9	−21	11	2044	6.39	57.24	<0.0001	<0.0001

Cluster threshold set at 100 voxels using *P* < 0.0001. Brain regions are based on the AAL atlas. AAL, automated anatomical labelling; MNI, Montreal Neurological Institute.

Fisher’s z-transformed correlation coefficient matrices corresponding to all 20 × 20 seeds revealed distinct connectivity patterns across groups. The pPVL group exhibited weakened intercortical correlations within the motor cortex and enhanced correlations within the central GM nuclei. The fPVL group showed less-pronounced alterations than the pPVL group (Fig. 1C).

**Figure 2:**
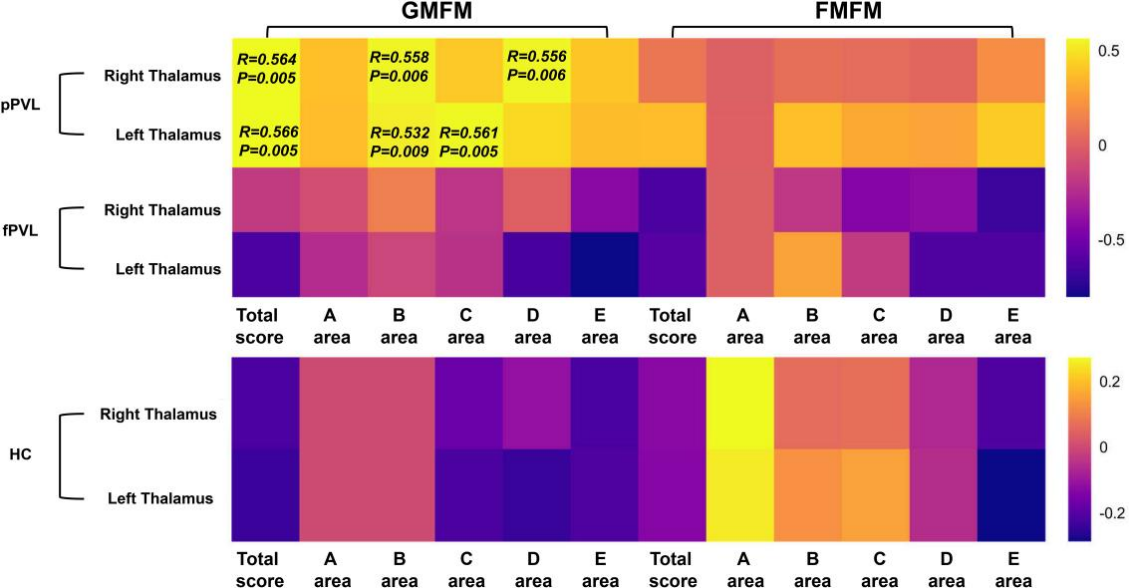
**Correlation between the GM volume and motor scores in the pPVL (*n* = 27), fPVL (*n* = 15) and HC (*n* = 38) groups.** The heatmap of the correlation matrix displays the values of the correlation coefficient obtained from partial correlation analysis, with age, sex and TIV as covariates. Colour mapping represents the correlation coefficients, with positive values represented in yellow and negative values in purple. *P* > 0.05 is shown as *P*-value in the graph as well as correlation coefficients.

In the combined PVL group, bilateral thalamic volumes positively correlated with GMFM total scores and GMFM B and C area scores. The right thalamic volume was positively correlated with the GMFM D area score. In the pPVL group, the right thalamic volume positively correlated with the GMFM total score and GMFM B and D area scores, whereas the left thalamic volume positively correlated with the GMFM total score and GMFM B and C area scores (*P* < 0.05; FWE-corrected). No significant correlations were found between FMFM scores and GM volume (Fig. 2).

### Graph theoretical analysis

**Figure 3:**
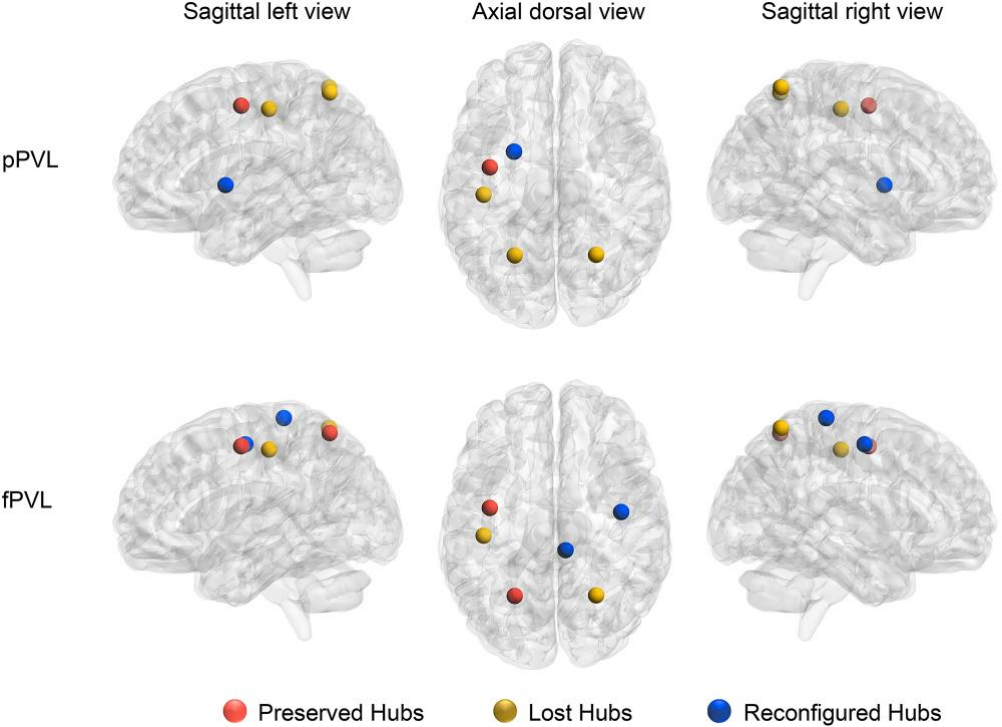
**Hubs reconfiguration in children with pPVL (*n* = 27) and those with fPVL (*n* = 15).** Red balls represent preserved hubs (hubs present in both the PVL and HC groups), yellow balls represent lost hubs (hubs present in the HC group but not in the PVL group), and blue balls represent reconfigured hubs (hubs present in the PVL group but not in the HC group). The nodal degree value of each of the 20 regions was calculated through graph theory (statistic values and *P*-values in Supplementary Table 5). Hubs were identified as brain regions having a node degree centrality of 1 SD higher than the network average. Hubs are represented on a three-dimensional brain template using the BrainNet toolbox.

The pPVL group had five hubs, whereas the fPVL group had six. Compared with the HC group, these hubs were classified as preserved, lost or reconfigured in the PVL groups. In the pPVL group, the lost hubs included the left postcentral gyrus and bilateral superior parietal lobule, whereas the left putamen was the reconfigured hub. In the fPVL group, the left postcentral gyrus and right superior parietal lobule had lost hubs, whereas the right PrG and right paracentral lobule had reconfigured hubs (Fig. 3).

**Figure 4:**
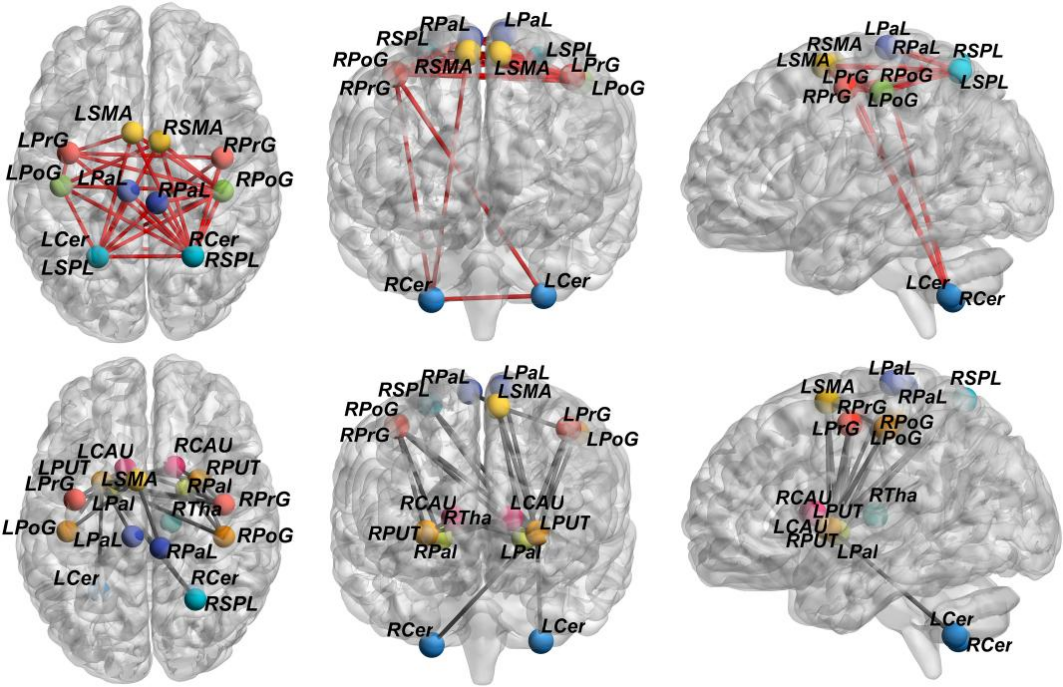
**Differences in the structural covariance network between the pPVL and HC groups.** Network-based statistic showed that compared with the HC group (*n* = 38), structural covariance in the pPVL groups (*n* = 27) was significantly weaker between the basal ganglia nuclei and other chosen regions, including the motor cortex and cerebellum (*P* < 0.001). Significantly stronger structural covariance in regions between the motor cortex and cerebellum (*P* < 0.001) was found in the pPVL group. Enhanced structural connections are represented by red lines, and weakened structural are represented by grey lines. LCAU, left caudate; LPal, left pallidum; LPaL, left paracentral lobule; LPoG, left postcentral gyrus; LPrG, left precentral gyrus; LPUT, left putamen; LSMA, left supplementary motor area; LSPL, left superior parietal lobule; LTha, left thalamus; LCer, left cerebellum; RCAU, right caudate; RCer, right cerebellum; RPal, right pallidum; RPaL, right paracentral lobule; RPoG, right postcentral gyrus; RPrG, right precentral gyrus; RPUT, right putamen; RSMA, right supplementary motor area; RSPL, right superior parietal lobule; RTha, right thalamus.

**Figure 5:**
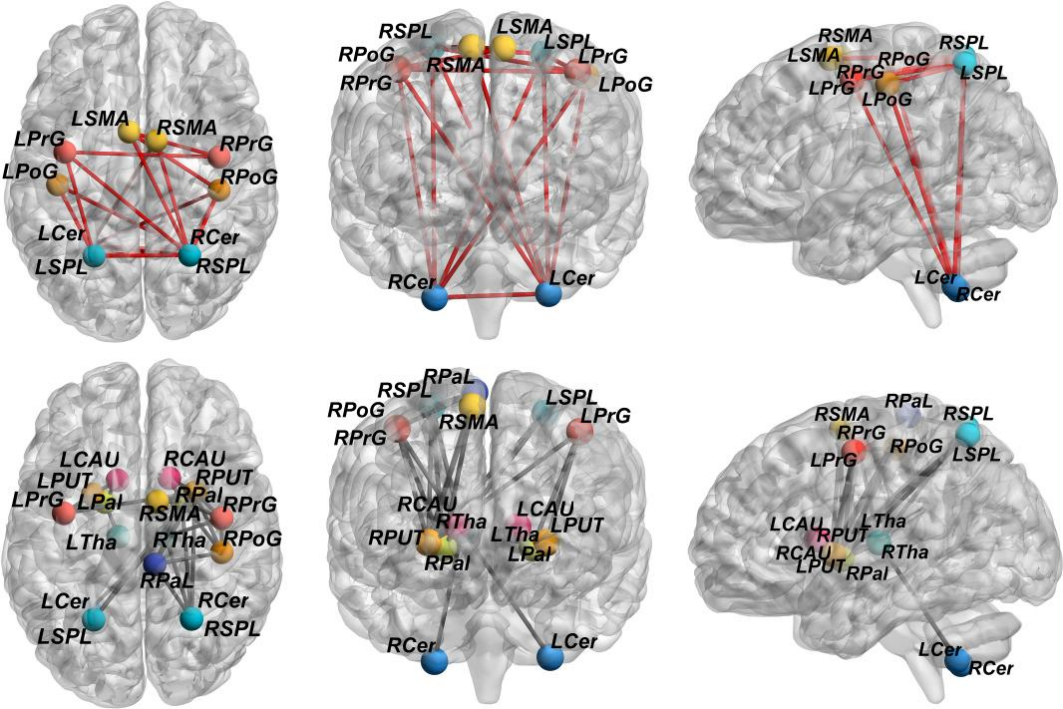
**Differences in the structural covariance network between the fPVL and HC groups.** Network-based statistic showed that structural covariance in the fPVL group (*n* = 15) was significantly weaker between the basal ganglia nuclei and other chosen regions, including motor cortex and cerebellum (*P* < 0.001), compared with the HC group (*n* = 38). The fPVL groups had significantly stronger structural covariance in regions between the motor cortex and cerebellum (*P* < 0.001). Enhanced structural connections are represented by red lines, and weakened structural are represented by grey lines. LCAU, left caudate; LPal, left pallidum; LPaL, left paracentral lobule; LPoG, left postcentral gyrus; LPrG, left precentral gyrus; LPUT, left putamen; LSMA, left supplementary motor area; LSPL, left superior parietal lobule; LTha, left thalamus; LCer, left cerebellum; RCAU, right caudate; RCer, right cerebellum; RPal, right pallidum; RPaL, right paracentral lobule; RPoG, right postcentral gyrus; RPrG, right precentral gyrus; RPUT, right putamen; RSMA, right supplementary motor area; RSPL, right superior parietal lobule; RTha, right thalamus.

**Figure 6:**
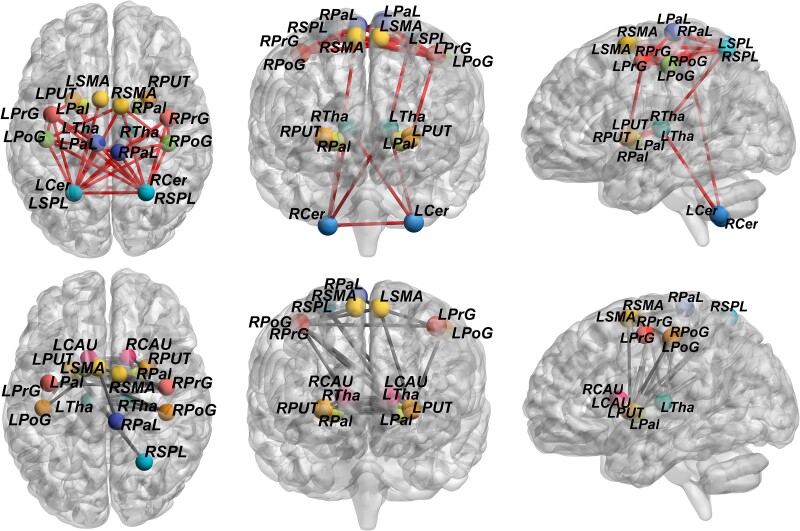
**Differences in the structural covariance network between the pPVL and fPVL groups.** Network-based statistic showed that compared with fPVL (*n* = 15), Structural covariance in the pPVL (*n* = 27) was significantly stronger between the basal ganglia nuclei and cerebellum (*P* < 0.001). Enhanced structural connections are represented by red lines, and weakened structural are represented by grey lines. LCAU, left caudate; LPal, left pallidum; LPaL, left paracentral lobule; LPoG, left postcentral gyrus; LPrG, left precentral gyrus; LPUT, left putamen; LSMA, left supplementary motor area; LSPL, left superior parietal lobule; LTha, left thalamus; LCer, left cerebellum; RCAU, right caudate; RCer, right cerebellum; RPal, right pallidum; RPaL, right paracentral lobule; RPoG, right postcentral gyrus; RPrG, right precentral gyrus; RPUT, right putamen; RSMA, right supplementary motor area; RSPL, right superior parietal lobule; RTha, right thalamus.

Both PVL groups exhibited whole-brain reconfiguration of structural connectivity. Compared with the HC group, both PVL groups showed significantly weaker connectivity between the central GM nuclei and other regions (including the motor cortex and cerebellum) and significantly stronger connectivity between the motor cortex and cerebellum (both *P* < 0.001; FWE-corrected; Figs 4–6).

Regarding global parameters, the pPVL group showed lower assortativity (*P* = 0.0300; FWE-corrected), longer shortest path length (*P* = 0.030; FWE-corrected), higher mean nodal betweenness centrality (*P* = 0.040; FWE-corrected) and higher mean edge betweenness centrality (*P* = 0.020; FWE-corrected) than the HC group. Both PVL groups exhibited increased lambda (*P* = 0.020; FWE-corrected) and decreased sigma (*P* = 0.0370; FWE-corrected) values compared with the HC group (Supplementary Table 2; Supplementary Fig. 2).

Significant differences in the nodal parameters were observed in both PVL groups. In the pPVL group, the bilateral caudate had a higher efficiency, whereas the left putamenhad a lower efficiency than in the HC group (Supplementary Table 3; Supplementary Fig. 3). In the fPVL group, the bilateral caudate and left putamen had higher nodal clustering coefficients (Supplementary Table 4; Supplementary Fig. 3) and nodal betweenness centrality (Supplementary Table 6; Supplementary Fig. 3) than in the HC group (*P* < 0.05; FWE-corrected).

The right postcentral lobule showed a lower local efficiency and clustering coefficient in the pPVL group than in the fPVL group. The right paracentral lobule in the pPVL group had lower nodal degree centrality (Supplementary Table 5; Supplementary Fig. 3), whereas the right caudate and left putamen had higher nodal degree centrality than the fPVL group (*P* < 0.05; FWE-corrected).

## Discussion

Motor behaviour relies on a network of core brain regions, including the primary motor cortex, premotor cortex, somatosensory and supplementary motor cortices, thalamus, putamen and cerebellum. In this study, we focused on these regions to explore the differential effects of PVL on the neural networks that underlie motor function in preterm and full-term infants.

Investigating alterations in structural covariation networks between children with pPVL and those with fPVL, four key findings emerged in this study: first, the voxel-based morphometry analysis revealed reduced thalamic volume in both PVL groups, with a positive correlation between the thalamic volume and GMFM scores; second, abnormal global network properties were mostly observed in the pPVL group; third, hub regions and regional nodal characteristics analysis showed reconfiguration of the basal ganglia nuclei in both PVL groups, particularly in the pPVL group; fourth, structural covariance of the thalamic and basal ganglia nuclei with the motor cortex and cerebellum decreased in both PVL groups, whereas structural covariance between the motor cortex and cerebellum increased.

In children with PVL, particularly those born prematurely, we observed a reduced thalamic volume and decreased structural covariance between the thalamus/basal ganglia and other motor pathways. We found that the distribution of foci in preterm infants was mostly bilateral, whereas in full-term infants, the foci were mostly unilaterally distributed, which may explain the differences in thalamic alterations between preterm and full-term infants. Our findings also support the notion that PVL-induced brain changes extend beyond the WM, affecting key regions involved in movement coordination within the motor network. The thalamus plays a crucial role in coordinating and modulating movements within the basal ganglia network,^21^ suggesting its importance in the pathogenesis of PVL.

Previous studies have shown that gestation time is linearly associated with brain morphometry during childhood development, with longer gestation being linked to greater brain volumes.^22^ Our findings were consistent with this observation. In addition, resting-state fMRI studies have demonstrated that preterm birth can reduce the complexity of the resting-state network covariance structure in newborns, even in the absence of structural damage.^23^ This supports the presence of abnormal structural networks in preterm infants in the present study, emphasizing the need to analyse abnormal nodes in movement-related regions. Thus, preterm infants may experience disruptions in brain connectivity that contribute to cognitive and motor dysfunctions.

### Abnormalities in the global topological properties of brain networks suggest incomplete neural network differentiation and integration

Abnormalities in the global topological properties of brain networks observed in both PVL groups compared with the HC group suggest an imbalance between neural network differentiation and integration due to brain injury. By computing the assortativity of different groups to measure the similarity of network connections, we found that pPVL was associated with lower assortativity, indicating a decreased correlation between motor nodes and a sparser, randomized network. The increased shortest path length in the pPVL group suggests longer information transfer paths between neural networks, potentially reflecting reduced network efficiency. However, the higher mean node and edge betweenness centrality in this group implies an increased information flow within the whole-brain motor network, possibly as a compensatory mechanism.

The human brain typically functions as a small-world network that combines low-energy consumption and high performance and is characterized by a high-clustering coefficient (facilitating local processing) and short characteristic path length (facilitating global processing). Sigma, an indicator of small worldness, and lambda, the standardized shortest path length, were used to measure the processing efficiency of the whole brain. In this study, sigma values decreased in the fPVL group and lambda values increased in the pPVL group, suggesting an imbalance in network differentiation and integration due to ischaemic–hypoxic damage and preterm birth.

These findings align with those of previous research demonstrating that severely injured brains exhibit less integrated and efficient network systems, as well as less integrated dorsal attention and frontoparietal, limbic and visual network systems. This suggests that perinatal injuries can lead to persistent network-level deviations that potentially contribute to neurological deficits.^24^

Recent studies have proposed that full-term brain network efficiency may serve as a sensitive biomarker for early CP detection because children with CP often display reduced network separation at multiple nodes.^17^

### Abnormalities in multiple node metrics suggest node reorganization in the whole-brain motor structure network

In children with pPVL, we observed increased nodal local efficiency in the bilateral caudate nuclei and decreased efficiency in the left putamen compared with HCs. Nodal local efficiency measures the fault tolerance within the network. This pattern suggests an imbalance in global integration, although local information processing may be enhanced through compensatory mechanisms.

Notably, the right postcentral gyrus showed a decreased clustering coefficient and local efficiency in the pPVL group compared with the fPVL group, indicating reduced information processing efficiency at these nodes. Conversely, in the fPVL group, the clustering coefficient decreased in the bilateral caudate nuclei compared with that in the HC group, suggesting increased connections between nodes and a shift towards a more regular network structure. Degree and betweenness centrality, measures of a node’s importance or influence in a network, were increased in the left putamen and right caudate nucleus in children with pPVL compared with children with fPVL, suggesting the presence of a compensatory network within the GM nuclei in the basal ganglia in children with pPVL. However, decreased degree centrality in the right paracentral lobule of children with pPVL compared with that in children with fPVL indicates reduced connectivity and information flow efficiency in the motor cortical areas.

### Structural covariance analysis suggests abnormal network connectivity patterns

Given the similar structural covariance results in both the PVL groups, we hypothesized that thalamic volume reduction reflects an altered motor network topology with changing connectivity patterns between nodes. Both PVL groups exhibited decreased connectivity between the thalamus/basal ganglia and motor cortex/cerebellum, whereas connectivity between the motor cortex and cerebellum increased. These findings suggest that the thalamus, as the most affected GM region, drives changes in peripheral network topography. Conversely, enhanced connectivity between the motor cortex and cerebellum may represent compensatory mechanisms that allow the motor network to adapt to individual physical activity demands.

In addition, electrophysiological studies have demonstrated that electrical stimulation of the cerebral cortex around ischaemic lesions can enhance neural activity, potentially improving the efficiency of neural processing in damaged network.^18^ The increased structural covariance between the motor cortex and cerebellum in children with PVL may reflect the brain’s utilization of alternative pathways to enhance information processing. Functional studies support this notion of developmental plasticity after childhood stroke, with increased connectivity between uninjured brain regions correlating with improved sensorimotor performance in children with unilateral CP.^19^ While the cerebellum is often spared from direct ischaemic–hypoxic injury, its neuromodulatory role may be crucial in the context of electrophysiological therapy.^25^

Previous research suggests that regions that are unaffected but at risk of atrophy are directly connected to the lesion through WM fibre bundles. Damage to these fibres may lead to reduced nutritional support, dendritic contraction and eventual atrophy of the connected regions. Although a mechanistic study is not feasible, WM disruption has been widely implicated in GM atrophy in numerous brain disorders.^26-28^

Compared with children with pPVL, those with fPVL showed less frequent thalamic involvement and weaker correlations between thalamic connectivity and motor performance. This suggests that thalamic involvement is less in full-term infants than in preterm infants, which may be related to the better development of the vascular endothelium in full-term infants, which confers greater resistance to ischaemic–hypoxic injury.

Hub analysis revealed that the left postcentral gyrus and bilateral parietal lobes had lost hubs, with the left basal ganglia acting as a reconfigured hub in children with pPVL. As pivotal components in the integration of brain functions, brain hubs facilitate the amalgamation of functionally specialized and anatomically disparate neural systems. This is evidenced by their propensity to establish long-range connections and topological positions within the brain, indicating that they mediate a substantial portion of the signal traffic. Our findings suggest that when thalamic involvement is severe, pPVL may be compensated for by activating the basal ganglia nuclei. Conversely, the fPVL showed minimal changes in global parameters, with increased nodal clustering efficiency only in the bilateral caudate nuclei and hub remodelling primarily localized to the motor cortex.

Preterm infants exhibited immature functional network patterns with high variability in multiple networks and a diminished correlation with motor scores. Regardless of the presence of PVL, preterm infants show enhanced structural connectivity within the cerebellum, which is associated with improved fine motor dexterity and visuomotor integration during adolescence. Dynamic functional connectivity also differs between preterm and full-term infants, with lower metastability and mean synchronization in preterm infants, suggesting specific dynamic alterations during the neonatal period in preterm infants. This alteration is associated with cognitive and motor outcomes at 18 months.^29^ Our results underscore the extensive alterations in the brain networks of preterm infants following ischaemic–hypoxic injury.

### Limitations

First, as a cross-sectional study, it could not account for potential changes in cerebral GM volume over time. Future longitudinal studies with follow-up data are needed to address these limitations. Second, we did not control for the extent of WM damage or disease severity in relation to the GM volume. More refined quantitative studies and targeted clinical assessments are required to further investigate these relationships. However, our focus was on identifying the reduced GM regions and characterizing differences in the injured motor network between the pPVL and fPVL groups, rather than examining the influence of WM or clinical factors. Third, the lack of a pure preterm infant control group (without PVL) made it difficult to determine whether the outcomes were due to the effects of preterm birth, PVL or their combination. Due to the challenge of accurately defining ‘preterm infants without motor abnormalities’ in early assessments and difficulty in deciding whether to exclude individuals with non-specific MRI features, we did not include such a cohort. Future studies should strive to develop more precise assessment protocols to address these issues. In this cohort, all patients with PVL were diagnosed with CP, as PVL is primarily caused by early cerebral ischaemia and hypoxia. However, CP can also arise from various other factors including abnormal brain development, infections, genetic factors, maternal health issues, prematurity, trauma, multiple pregnancies, blood supply problems and immune system abnormalities. This study did not address the differences in aetiology between preterm and full-term infants. Future studies should investigate the mechanisms underlying the development of CP in the context of these diverse aetiological factors.

In summary, children with pPVL and fPVL showed abnormal motor network patterns, with children with pPVL displaying more extensive central GM nuclei atrophy and a wider distribution of abnormalities. In contrast, network remodelling in children with fPVL was more localized to the motor cortex. Both groups showed decreased connectivity between the central GM nuclei and other regions and increased connectivity between the motor cortex and cerebellar hemispheres. These findings suggest differences in abnormal connectivity between children with pPVL and fPVL, which may indicate a pattern of coexisting impairment and compensation at different stages. This study highlights the potential of structural covariance patterns for monitoring brain development and advancing our understanding of aberrant brain development in children with PVL.

## Supplementary Material

fcae405_Supplementary_Data

## Data Availability

Data supporting the findings of this study are available upon request from the corresponding authors.
