# Supplementary material for: Structural covariance alterations reveal motor damage in periventricular leukomalacia
Source: Brain Commun. 2024 Nov 12;6(6):fcae405. doi: 10.1093/braincomms/fcae405 (PMC11589463; doi:10.1093/braincomms/fcae405)
Supplement: fcae405_Supplementary_Data [file fcae405_supplementary_data.pdf]

Supplementary material

Additional detail for calculating the graph theory network-based properties.

Global Efficiency measures the overall efficiency of information transfer across the network. It is the average of the inverse of the shortest path lengths between all pairs of nodes. For each pair of nodes i and j, compute the shortest path length d_{ij}. The global efficiency is calculated as:

$$E_{global} = \frac{1}{N(N-1)} \sum_{i \neq j} \frac{1}{d_{ij}}$$

where NNN is the total number of nodes in the network.

Local Efficiency quantifies the efficiency of information transfer within the neighborhood of a node. Local efficiency is calculated as:

$$E_{global} = \frac{1}{N(N-1)} \sum_{i \neq j} \frac{1}{d_{ij}} E_{loc}(i) = \frac{1}{\mathrm{n}_i(n_i - 1)} \sum_{j,k \in N(i)} \frac{1}{d_{jk}}$$

where N(i) is the neighborhood of node i, n_i is the number of nodes in the neighborhood, and d_{jk} is the shortest path length between nodes j and k in the neighborhood.

Assortativity measures the similarity of connections between nodes in a network, often reflecting the correlation of node attributes. The assortativity coefficient r is computed as:

$$r = \frac{\sum_i (k_i - \bar{k})(k_j - \bar{k}) P(k_i, k_j)}{\sqrt{\sum_i (k_i - \bar{k})^2 P(k_i) \sum_j (k_j - \bar{k})^2 P(k_j)}}$$

where k_i and k_j are the degrees of nodes i and j, $\bar{\mathrm{K}}$ is the average degree, and $P(k_i,k_j)$ is the probability of nodes with degrees k_i and k_j being connected.

Clustering Coefficient reflects the density of connections within a node's neighborhood. The clustering coefficient C_i for node i is given by:

$$C_i = \frac{2T_i}{k_i(k_i - 1)}$$

where T_i is the number of triangles in the neighborhood of node i, and k_i is the degree of node i. The overall network clustering coefficient C is the average of all node clustering coefficients:

$$C = \frac{1}{N} \sum_i C_i$$

Shortest Path Length is the length of the shortest connection between two nodes. Shortest path

lengths can be computed using algorithms such as Dijkstra's or Floyd-Warshall. The characteristic path length L is the average shortest path length between all pairs of nodes:

$$L = \frac{1}{N(N-1)} \sum_{i \neq j} d_{ij}$$

Small-Worldness Index

Small-world attributes (γ, λ, and σ) indicate the degree of small-world organization, which reflects an optimal balance of integration and segregation for a network. In the definition of small-world properties, normalized clustering coefficient γ = C_real/C_random, normalized characteristic path length λ = L_real/L_random, and small-world coefficient σ = γ/λ, the small-worldness is satisfied if σ > 1; γ > 1 and λ≈ 1.

Nodal Local Efficiency is the local efficiency of a specific node, reflecting the efficiency of information transfer within its neighborhood. Computed similarly to local efficiency but specifically for each node's neighborhood.

Nodal Degree Centrality is the number of direct connections a node has, indicating its direct connectivity capability. The degree centrality k_i of node i is:

$$k_i = \sum_j A_{ij}$$

where A_{ij} is the weight of the edge between nodes i and j (0 or 1).

Nodal Betweenness Centrality measures the extent to which a node lies on the shortest paths between other nodes. The betweenness centrality BC_i of node i is:

$$BC_i = \sum_{s \neq i \neq t} \frac{\sigma_{st}(i)}{\sigma_{st}}$$

where σ_{st} is the total number of shortest paths between nodes s and t, and σ_{st} (i) is the number of those paths that pass through node i.

Nodal Clustering Coefficient measures the clustering within the neighborhood of a specific node. For node i, the clustering coefficient C_i is:

$$C_i = \frac{2T_i}{k_i(k_i - 1)}$$

where T_i is the number of triangles in the neighborhood of node i, and k_i is the degree of node i.

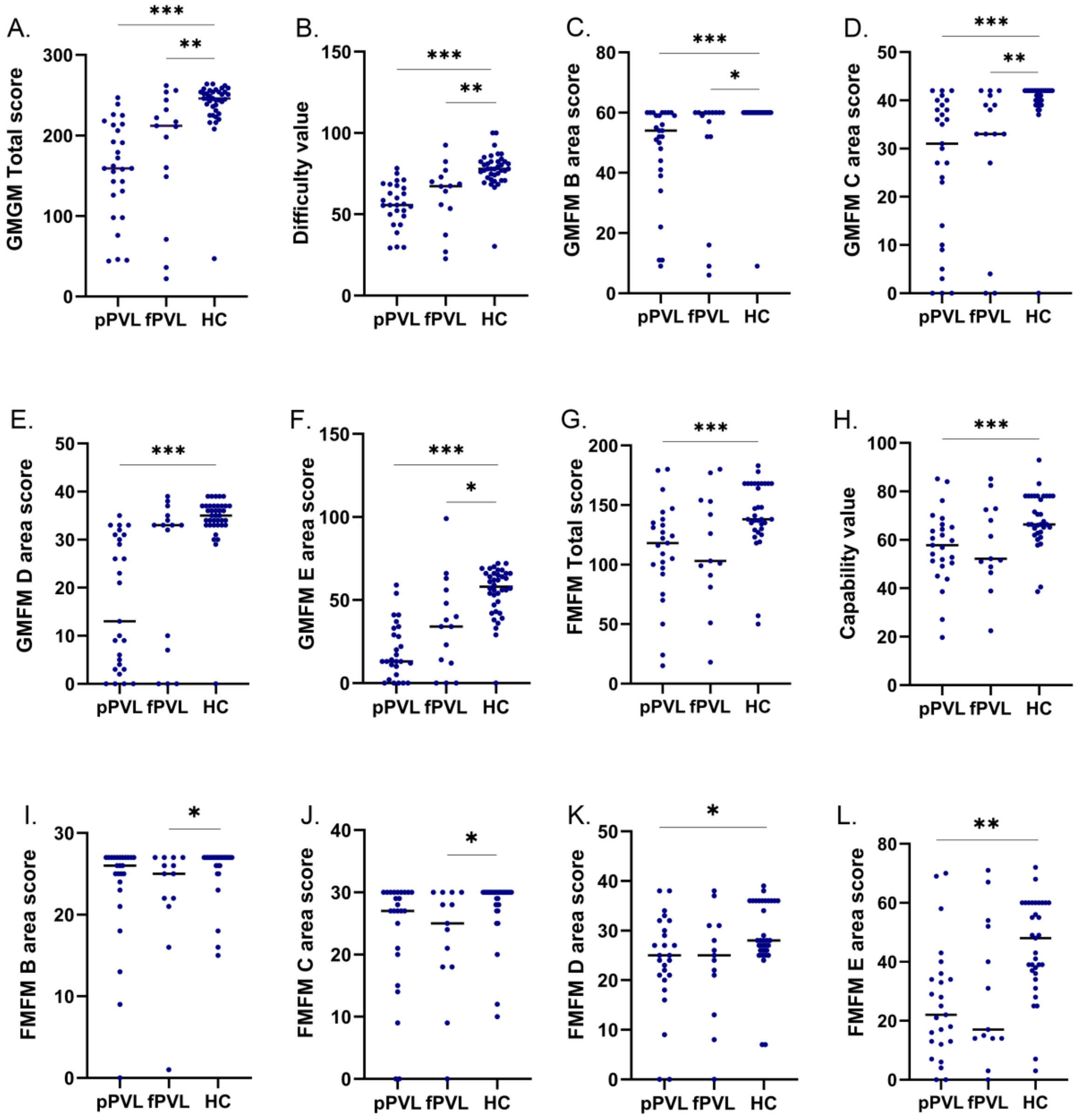

Supplemental Figure 1 Comparison of GMFM and FMFM motor scores among pPVL (n=27), fPVL (n=15) and HC group (n=38).

Horizontal lines denote the median values. Asterisks indicate significant P-values by post hoc comparisons of ANOVAs. ***=p<0.001; **=p<0.01; *=p<0.05; pPVL=preterm Periventricular Leukomalacia; fPVL=full-term Periventricular Leukomalacia; HC=Health Control; GMFM=gross motor function measure; FMFM=fine motor function measure.

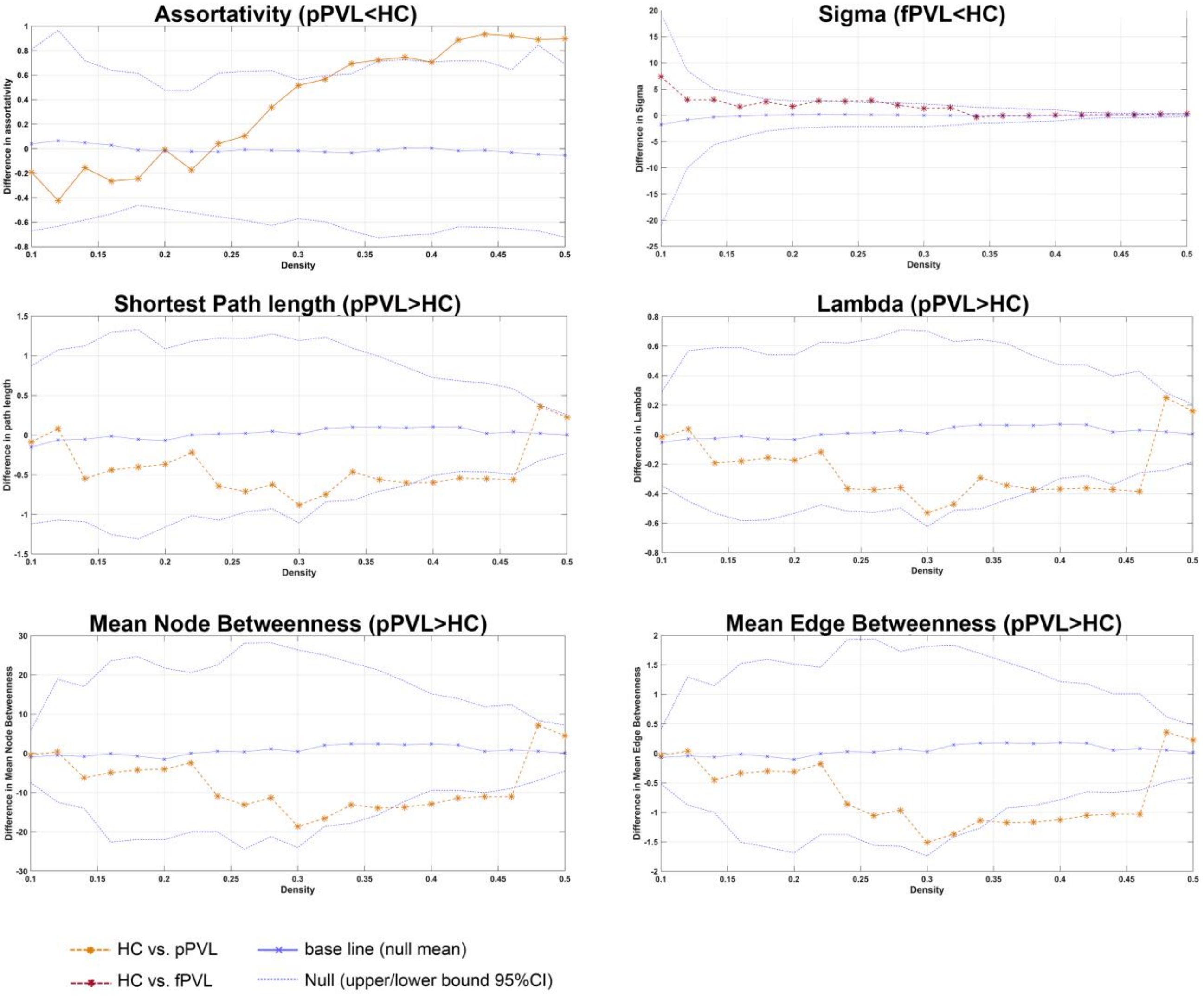

Supplemental Figure 2 Global differences of attributes in the structural covariance network among the pPVL (n=27), fPVL (n=15) and HC group (n=38). The purple straight line represented the mean structural covariance in the randomized data, averaged across 5,000 randomizations. The area bounded by the upper and lower dotted lines denoted 95% confidence intervals across the 5,000 randomizations. One tail student's *t*-tests were performed at each density value, comparing global measures in patients (pPVL or fPVL) to controls. The orange (or red dots) outside the 95% confidence interval represented significant differences p FDR < 0.05. FDR=false discovery rate. pPVL=preterm Periventricular Leukomalacia; fPVL=full-term Periventricular Leukomalacia; HC=Health Control

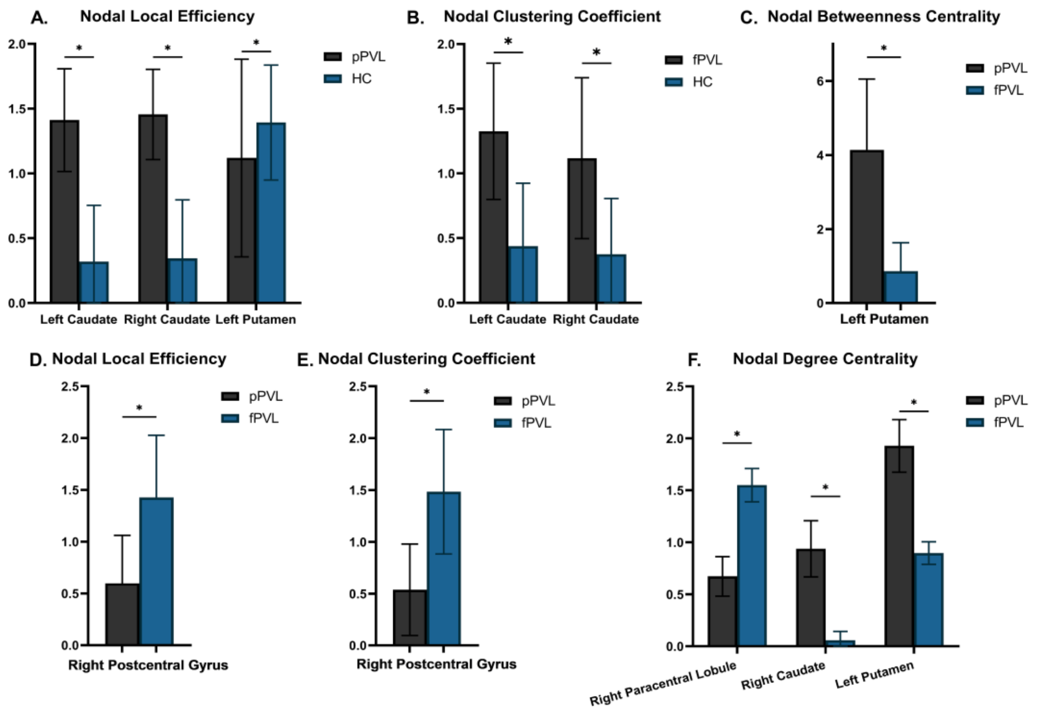

Supplemental Figure 3 Graph analysis of nodal properties in pPVL (n=27), fPVL (n=15) and HC group (n=38).

The bar chart was used to display the nodal properties, and the horizontal short lines represent standard deviations. Permutation test was applied with P value displayed in Supplementary Table 3-6. *=p<0.05. pPVL = preterm Periventricular Leukomalacia; fPVL = full-term Periventricular Leukomalacia; HC = Health Control.

Supplemental Table1. Perinatal high-risk events and motor assessment of the sample

	PVL (n=42)	HC (n=38)	Statistics (PVL/HC) (c^2/t/Z, p-value)	pPVL (n=27)	fPVL (n=15)	Statistics (pPVL/fPVL/HC)(c^2/ANOVA, p-value)
**Perinatal adverse events**						
Asphyxia, N [%]	13[31.0%]	-	-	9[33.3%]	4[26.7%]	-
Intrauterine infections, N [%]	2[4.8%]	-	-	2[7.4%]	0	-
Intracranial hemorrhage, N [%]	6[14.3%]	-	-	6[22.2%]	0	-
Neonatal jaundice, N [%]	11[26.2%]	-	-	9[33.3%]	2[13.3%]	-
neonatal HIE, N [%]	6[14.3%]	-	-	5[18.5%]	1[6.7%]	-
NRDS, N [%]	8[19.0%]	-	-	8[29.6%]	0	-
Neonatal pneumonia, N [%]	3[7.1%]	-	-	3[11.1%]	0	-
Neonatal sepsis, N [%]	3[7.1%]	-	-	2[7.4%]	1[6.7%]	-
Neonatal meningitis, N [%]	1[2.4%]	-	-	1[3.7%]	0	-
Neonatal Anaemia, N [%]	1[2.4%]	-	-	1[3.7%]	0	-
**Maternal complications during pregnancy**						
Hypertension in pregnancy, N [%]	2[4.8%]	-	-	2[7.4%]	0	-
Gestational diabetes, N [%]	2[4.8%]	-	-	2[7.4%]	0	-
Placenta previa, N [%]	1[2.4%]	-	-	1[3.7%]	0	-
**GMFM**						
GMFM total score, mean[s.d.]	166.93[67.73]	238.34[35.08]	t=-6.001, p<0.001***	159.86[58.88]	182.69[84.82]	F=17.896, p<0.001***
GMFM (%), mean[s.d.]	65.33[26.27]	91.79[13.15]	t=-5.775, p<0.001***	62.72[23.06]	71.15[32.62]	F=16.501, p<0.001***
Difficulty Value, mean[s.d.]	57.70[16.15]	77.79[11.09]	t=-6.483, p<0.001***	56.22[13.36]	61.28[21.79]	F=21.030, p<0.001***
A area, mean[s.d.]	48.10[7.45]	50.66[2.11]	t=-2.136, p=0.038*	48.83[4.98]	46.46[11.30]	F=2.918, p=0.060
B area, mean[s.d.]	47.88[17.51]	58.66[8.27]	t=-3.572, p=0.001**	48.00[15.85]	47.62[21.47]	F=5.910, p=0.004**
C area, mean[s.d.]	27.50[15.14]	39.79[6.79]	t=-4.758, p<0.001***	26.69[14.78]	29.31[16.38]	F=10.719, p<0.001***
D area, mean[s.d.]	19.31[14.50]	34.16[6.25]	t=-6.046, p<0.001***	16.79[13.35]	24.92[15.89]	F=20.297, p<0.001***
E area, mean[s.d.]	24.95[22.67]	55.08[14.87]	t=-7.089, p<0.001***	19.76[16.75]	36.54[29.85]	F=29.716, p<0.001***
**FMFM**						
FMFM total score, mean[s.d.]	112.42[43.27]	134.63[30.79]	t=-2.357, p=0.022*	110.65[41.21]	116.25[49.12]	F=2.440, p=0.096
Ability Value, mean[s.d.]	57.34[16.02]	65.93[11.59]	t=-2.443, p=0.018*	58.96[18.35]	56.59[15.15]	F=2.660, p=0.078
A area, mean[s.d.]	14.97[0.16]	14.79[1.02]	t=0.867, p=0.395	15.00[0.00]	14.92[0.29]	F=0.646, p=0.528
B area, mean[s.d.]	23.03[6.78]	25.25[3.60]	t=-1.477, p=0.145	23.23[6.56]	22.58[7.53]	F=1.125, p=0.332
C area, mean[s.d.]	23.32[9.09]	27.08[5.51]	t=-2.031, p=0.047**	23.58[9.06]	22.75[9.55]	F=1.686, p=0.194
D area, mean[s.d.]	23.66[10.07]	27.38[7.76]	t=-1.541, p=0.129	23.38[9.83]	24.25[10.99]	F=1.204, p=0.307
E area, mean[s.d.]	27.37[20.90]	41.21[17.01]	t=-2.722, p=0.008**	25.46[19.12]	31.50[24.71]	F=4.085, p=0.022*

Note. IVF=in vitro fertilization; NRDS=neonatal respiratory distress syndrome； HIE=hypoxic-ischemic encephalopathy； PVL=periventricular leukomalacia； HC=health control； pPVL=premature-PVL； fPVL=full-term-PVL； GMFM=gross motor function measure; FMFM=fine motor function measure; ANOVA=analysis of variance; s.d.=standard deviation; c^2=chi square test; M=median; ***=p<0.001; **=p<0.01; *=p<0.05

Supplemental Table2. Group comparison of structural network parameters

	pPVL (n=27)	fPVL (n=15)	HC (n=38)	pPVL -HC	fPVL-HC	pPVL -fPVL
	Mean(95%CI)				*p*	
Assortativity	0.168(0.043, 0.292)	0.377(0.317, 0.438)	0.525(0.421, 0.629)	0.03*	0.344	0.175
Global Efficiency	0.502(0.408, 0.596)	0.501(0.409, 0.593)	0.412(0.318, 0.506)	0.086	0.095	0.408
Shortest Path Length	1.671(1.556, 1.786)	1.524(1.260, 1.789)	1.247(1.148, 1.347)	0.03*	0.25	0.452
MNBC	11.85(9.434, 14.270)	8.80(5.307, 10.30)	3.876(1.945, 5.810)	0.04*	0.26	0.486
MEBC	1.310(1.069, 1.552)	1.103(0.680, 1.526)	0.622(0.443, 0.801)	0.02*	0.218	0.457
Lambda	0.932(0.836, 1.028)	0.814(0.610, 1.078)	0.694(0.604, 0.785)	0.02*	0.272	0.402
Sigma	2.867(1.629, 4.105)	2.141(1.326, 2.955)	3.805(2.194, 5.417)	0.29	0.037*	0.429
Gamma	2.170(1.727, 2.614)	1.756(1.457, 2.056)	2.278(1.671, 2.885)	0.48	0.113	0.4

*=p<0.05; pPVL=preterm Periventricular Leukomalacia; fPVL=full-term Periventricular Leukomalacia; HC=Health Control; MNBC=Mean Nodal Betweenness Centrality; MEBC= Mean Edge Betweenness Centrality

Supplemental Table3. Group comparison of Nodal local efficiency

		pPVL (n=27)	fPVL (n=15)	HC (n=38)	pPVL -HC	fPVL-HC	pPVL - fPVL
		Mean(95%CI)				*p*	
PrG	L	0.639(0.577, 0.702)	0.201(0.134, 0.268)	0.542(0.493, 0.591)	0.848	0.548	0.227
	R	0.602(0.535, 0.670)	0.273(0.222, 0.324)	0.500(0.443, 0.557)	0.868	0.064	0.335
SMA	L	0.567(0.509, 0.626)	0.526(0.526, 0.526)	0.439(0.388, 0.491)	0.631	0.899	0.335
	R	0.433(0.374, 0.493)	0.526(0.526, 0.526)	0.461(0.396, 0.527)	0.605	0.629	0.466
PoG	L	0.568(0.496, 0.639)	0.241(0.189, 0.292)	0.569(0.517, 0.621)	0.043	0.366	0.291
	R	0.233(0.149, 0.316)	0.549(0.412, 0.585)	0.425(0.374, 0.477)	0.102	0.834	0.035*
SPL	L	0.464(0.394, 0.534)	0.576(0.567, 0.586)	0.566(0.517, 0.616)	0.132	0.956	0.203
	R	0.470(0.397, 0.543)	0.527(0.466, 0.588)	0.550(0.500, 0.600)	0.233	0.466	0.31
PaL	L	0.522(0.466, 0.578)	0.242(0.202, 0.282)	0.448(0.363, 0.534)	0.596	0.041	0.303
	R	0.274(0.218, 0.330)	0.168(0.129, 0.207)	0.407(0.312, 0.503)	0.232	0.043	0.249
Cau	L	0.550(0.498, 0.601)	0.214(0.202, 0.276)	0.132(0.083, 0.180)	0.023*	0.658	0.432
	R	0.522(0.481, 0.563)	0.217(0.202, 0.276)	0.141(0.085, 0.197)	0.020*	0.66	0.114
Put	L	0.710(0.646, 0.775)	0.526(0.526, 0.526)	0.223(0.178, 0.268)	<0.001***	0.275	0.399
	R	0.559(0.511, 0.607)	0.526(0.526, 0.526)	0.210(0.169, 0.251)	0.09	0.105	0.113
Pal	L	0.559(0.493, 0.625)	0.526(0.526, 0.526)	0.245(0.187, 0.303)	0.102	0.08	0.406
	R	0.518(0.472, 0.564)	0.526(0.526, 0.526)	0.247(0.188, 0.374)	0.313	0.425	0.052
Tha	L	0.339(0.250, 0.428)	0.230(0.210, 0.278)	0.282(0.189, 0.374)	0.909	0.499	0.389
	R	0.238(0.179, 0.298)	0.240(0.210, 0.278)	0.231(0.158, 0.303)	0.783	0.486	0.345
Cer	L	0.466(0.390, 0.541)	0.526(0.526, 0.526)	0.465(0.414, 0.515)	0.661	0.349	0.318
	R	0.496(0.418, 0.574)	0.526(0.526, 0.526)	0.455(0.398, 0.511)	0.952	0.37	0.32

*=p<0.05; ***=p<0.001; pPVL=preterm Periventricular Leukomalacia; fPVL=full-term Periventricular Leukomalacia; HC=Health Control; Cau = Caudate; Pal = Pallidum; PaL = Paracentral Lobule; PoG = Postcentral Gyrus; PrG = Precentral Gyrus; Put = Putamen; SMA = Supplementary Motor Area; SPL = Superior Parietal Lobule; Tha = Thalamus; Cer=Cerebellum; L=Left; R=Right

Supplemental Table4. Group comparison of Nodal clustering Coefficient

		pPVL(n=27)	fPVL(n=15)	HC(n=38)	pPVL -HC	fPVL -HC	pPVL -fPVL
			Mean(95%CI)			*p*	
PrG	L	0.843(0.716, 0.970)	1.096(0.723, 1.469)	1.083(1.016, 1.150)	0.633	0.177	0.186
	R	0.972(0.794, 1.151)	1.245(0.919, 1.571)	1.196(0.954, 1.438)	0.633	0.161	0.251
SMA	L	1.159(0.954, 1.365)	1.217(0.960, 1.474)	0.986(0.777, 1.950)	0.644	0.174	0.443
	R	1.164(0.892, 1.435)	0.966(0.807, 1.125)	1.177(0.974, 1.379)	0.767	0.398	0.463
PoG	L	1.048(0.774, 1.321)	1.291(1.147, 1.434)	1.034(0.914, 1.153)	0.767	0.334	0.244
	R	0.608(0.357, 0.860)	1.442(1.193, 1.691)	1.295(1.100, 1.491)	0.766	0.106	0.019*
SPL	L	0.799(0.540, 1.059)	1.077(0.969, 1.184)	1.136(1.005, 1.268)	0.644	0.368	0.299
	R	0.560(0.344, 0.776)	1.359(1.123, 1.595)	1.206(1.031, 1.381)	0.644	0.37	0.342
PaL	L	1.084(0.951, 1.217)	1.351(1.147, 1.555)	0.842(0.638, 1.046)	0.766	0.365	0.437
	R	1.092(0.804, 1.380)	1.143(1.020, 1.266)	0.826(0.545, 1.107)	0.766	0.408	0.323
Cau	L	0.502(0.300, 0.704)	1.326(1.086, 1.566)	0.437(0.216, 0.659)	0.960	0.013*	0.47
	R	0.605(0.463, 0.748)	1.118(0.835, 1.401)	0.375(0.179, 0.571)	0.960	0.018*	0.808
Put	L	0.958(0.602, 1.315)	0.996(0.759, 1.235)	1.462(1.229, 1.694)	0.300	0.266	0.333
	R	1.302(0.936, 1.668)	0.768(0.579, 0.958)	1.493(1.275, 1.711)	0.300	0.248	0.194
Pal	L	1.285(0.907, 1.662)	0.838(0.595, 1.082)	1.349(1.074, 1.625)	0.633	0.148	0.414
	R	1.467(1.124, 1.810)	0.728(0.548, 0.909)	1.488(1.269, 1.708)	0.766	0.207	0.098
Tha	L	0.288(0.044, 0.531)	0.414(0.173, 0.655)	0.353(0.138, 0.569)	0.767	0.527	0.373
	R	0.462(0.217, 0.708)	0.339(0.198, 0.480)	0.394(0.155, 0.632)	0.767	0.578	0.386
Cer	L	1.014(0.7419, 1.286)	0.598(0.323, 0.873)	1.027(0.851, 1.202)	0.766	0.618	0.359
	R	0.783(0.550, 1.016)	0.682(0.378, 0.987)	1.073(0.811, 1.334)	0.633	0.622	0.343

*=p<0.05; pPVL=preterm Periventricular Leukomalacia; fPVL=full-term Periventricular Leukomalacia; HC=Health Control; Cau = Caudate; Pal = Pallidum; PaL = Paracentral Lobule; PoG = Postcentral Gyrus; PrG = Precentral Gyrus; Put = Putamen; SMA = Supplementary Motor Area; SPL = Superior Parietal Lobule; Tha = Thalamus; Cer=Cerebellum; L=Left; R=Right

Supplemental Table5 Group comparison of Nodal degree centrality

		pPVL(n=27)	fPVL(n=15)	HC(n=38)	pPVL-HC	fPVL-HC	pPVL-fPVL
		Mean(95%CI)				*p*	
PrG	L	8.619(7.148, 10.09)	10.240(8.682, 11.790)	8.523(7.677, 9.369)	0.63	0.548	0.091
	R	8.762(7.102, 10.42)	8.238(6.843, 9.633)	7.205(6.042, 8.367)	0.44	0.064	0.469
SMA	L	7.143(6.185, 8.101)	4.619(3.394, 5.844)	5.409(4.516, 6.302)	0.39	0.435	0.197
	R	3.905(3.042, 4.767)	6.286(4.663, 7.909)	6.455(5.238, 7.671)	0.23	0.629	0.278
PoG	L	7.81(6.072, 9.547)	8.667(7.269, 10.060)	9.500(8.600, 10.400)	0.09	0.366	0.18
	R	5.952(3.546, 8.359)	7.048(5.725, 8.371)	8.227(7.255, 9.200)	0.13	0.834	0.203
SPL	L	8.571(8.271, 8.871)	9.810(8.366, 11.250)	9.364(8.516, 10.210)	0.11	0.956	0.058
	R	8.857(8.730, 8.984)	8.048(6.975, 9.120)	8.818(7.933, 9.703)	0.1	0.466	0.105
PaL	L	6.286(5.319, 7.253)	8.000(6.737, 9.263)	6.750(5.368, 8.132)	0.58	0.041	0.193
	R	4.048(3.121, 4.975)	8.619(7.148, 10.090)	5.864(4.222, 7.505)	0.41	0.043	0.048*
Cau	L	4.952(4.351, 5.554)	2.714(2.173, 3.256)	1.364(1.118, 1.610)	0.11	0.658	0.177
	R	4.810(4.340, 5.279)	0.476(0.135, 0.817)	1.341(1.087, 1.594)	0.13	0.66	0.036*
Put	L	10.520(8.983, 12.060)	5.048(4.077, 6.018)	8.977(8.703, 9.251)	0.01	0.987	0.011*
	R	7.571(6.379, 8.763)	5.762(5.345, 6.179)	8.568(8.328, 8.808)	0.08	0.802	0.091
Pal	L	8.952(7.488, 10.420)	5.048(4.056, 6.039)	5.136(4.751, 5.522)	0.18	0.913	0.088
	R	4.381(5.421, 7.341)	3.905(2.634, 5.175)	3.182(2.771, 3.593)	0.3	0.634	0.08
Tha	L	3.048(2.143, 3.952)	4.667(3.635, 5.698)	3.523(2.281, 4.76)	0.65	0.499	0.345
	R	2.524(1.903, 3.145)	2.000(0.922, 3.070)	2.000(1.488, 2.512)	0.6	0.486	0.276
Cer	L	1.905(1.475, 2.334)	2.476(2.202, 2.750)	2.318(1.527, 3.110)	0.16	0.349	0.415
	R	2.381(1.561, 3.201)	3.333(2.478, 4.189)	5.841(4.911, 6.770)	0.58	0.37	0.403

*=p<0.05; pPVL=preterm Periventricular Leukomalacia; fPVL=full-term Periventricular Leukomalacia; HC=Health Control; Cau = Caudate; Pal = Pallidum; PaL = Paracentral Lobule; PoG = Postcentral Gyrus; PrG = Precentral Gyrus; Put = Putamen; SMA = Supplementary Motor Area; SPL = Superior Parietal Lobule; Tha = Thalamus; Cer=Cerebellum; L=Left; R=Right. The visualization of node reconfiguration is presented in Figure 3.

Supplemental Table6 Group comparison of Nodal betweenness centrality

		pPVL(n=27)	fPVL(n=15)	HC(n=38)	pPVL-HC	fPVL-HC	pPVL-fPVL
			Mean(95%CI)			*p*	
PrG	L	31.710(23.060, 40.350)	49.880(40.140, 59.620)	35.265(32.059, 38.472)	0.06	0.548	0.246
	R	26.610(20.100, 33.120)	17.360(10.120, 24.600)	23.443(22.226, 29.111)	0.2	0.064	0.386
SMA	L	8.307(6.311, 10.300)	5.836(3.796, 6.876)	8.346(8.043, 8.650)	0.13	0.948	0.157
	R	3.405(3.180, 3.631)	2.576(1.536, 3.616)	2.225(1.373, 3.077)	0.26	0.629	0.226
PoG	L	33.050(20.590, 45.510)	26.290(17.520, 38.070)	30.940(30.263, 31.610)	0.26	0.366	0.444
	R	14.290(8.757, 19.830)	6.118(3.816, 8.419)	13.103(12.211, 13.996)	0.18	0.834	0.571
SPL	L	29.490(15.100, 43.880)	26.390(20.150, 28.630)	24.903(22.748, 27.059)	0.55	0.956	0.132
	R	6.155(2.199, 10.110)	10.370(7.998, 12.740)	6.702(4.178, 8.225)	0.29	0.466	0.41
PaL	L	9.320(5.635, 13.010)	11.410(7.266, 15.560)	14.940(10.836, 34.720)	0.47	0.041	0.205
	R	8.453(8.252, 8.654)	17.140(11.040, 23.250)	10.099(10.013, 11.212)	0.67	0.043	0.353
Cau	L	1.582(0.416, 2.747)	0.353(-0.282, 0.988)	4.584(1.270, 7.898)	0.64	0.658	0.465
	R	1.344(1.123, 1.565)	0.850(-0.623, 1.624)	3.275(0.9417, 5.608)	0.6	0.66	0.597
Put	L	55.790(46.550, 65.040)	10.660(7.152, 14.160)	50.830(50.043, 51.704)	0.08	0.568	0.005**
	R	17.360(13.010, 21.710)	15.151(11.613, 18.689)	12.451(12.277, 17.179)	0.09	0.642	0.161
Pal	L	37.300(26.770, 47.830)	18.250(7.028, 29.480)	34.497(32.589, 38.405)	0.14	0.821	0.359
	R	6.588(4.621, 8.556)	8.080(5.729, 9.430)	6.2778（6.087， 6.6427）	0.6	0.305	0.508
Tha	L	5.444(1.832, 9.057)	6.350(2.190, 7.520)	7.816（-2.795, 18.430)	0.85	0.499	0.058
	R	1.145(0.552, 1.737)	4.517(1.777, 7.257)	3.603(-1.679, 8.883)	0.79	0.486	0.692
Cer	L	1.714(-1.862, 5.290)	4.140(0.357, 7.923)	2.209(-0.087, 4.505)	0.4	0.349	0.748
	R	1.125(0.240, 2.009)	1.082(0.926, 1.239)	0.0158(-0.017, 0.0499)	0.13	0.37	0.685

*=p<0.05; **=p<0.01; pPVL=preterm Periventricular Leukomalacia; fPVL=full-term Periventricular Leukomalacia; HC=Health Control; Cau = Caudate; Pal = Pallidum; PaL = Paracentral Lobule; PoG = Postcentral Gyrus; PrG = Precentral Gyrus; Put = Putamen; SMA = Supplementary Motor Area; SPL = Superior Parietal Lobule; Tha = Thalamus; Cer=Cerebellum; L=Left; R=Right

The analytical software involved in this study are listed here:

MATLAB R2019a(The MathWorks Inc., Natick, MA, USA)(https://ww2.mathworks.cn/help/matlab/release-notes-R2019a.html?requestedDomain=cn)
SPM12; The Wellcome Centre for Human Neuroimaging, UCL Queen Square Institute of Neurology, London, UK; www.fil.ion.ucl.ac.uk/spm
SPSS (version 20.0; IBM Corp., Armonk, NY)(https://www.ibm.com/support/pages/downloading-ibm-spss-statistics-20)
MarsBar (http://marsbar.sourceforge.net)
Graph Analysis Toolbox (GAT) (http://ncnl.stanford.edu/tools. html)
Network-based statistic (NBS) (http://www.nitrc.org/projects/nbs/)
G*Power software (https://www.psychologie.hhu.de/arbeitsgruppen/allgemeine-psychologie-und-arbeitspsychologie/gpower)
